# Identification of Direct Activator of Adenosine Monophosphate-Activated Protein Kinase (AMPK) by Structure-Based Virtual Screening and Molecular Docking Approach

**DOI:** 10.3390/ijms18071408

**Published:** 2017-06-30

**Authors:** Tonghui Huang, Jie Sun, Shanshan Zhou, Jian Gao, Yi Liu

**Affiliations:** Jiangsu Key Laboratory of New Drug Research and Clinical Pharmacy, School of Pharmacy, Xuzhou Medical University, Xuzhou 221004, China; jxbp0812@163.com (J.S.); ZSS1991530@163.com (S.Z.); gaojian@xzhmu.edu.cn (J.G.)

**Keywords:** Adenosine 5′-monophosphate-activated protein kinase, virtual screening, molecular docking, selective activator

## Abstract

Adenosine monophosphate-activated protein kinase (AMPK) plays a critical role in the regulation of energy metabolism and has been targeted for drug development of therapeutic intervention in Type II diabetes and related diseases. Recently, there has been renewed interest in the development of direct β1-selective AMPK activators to treat patients with diabetic nephropathy. To investigate the details of AMPK domain structure, sequence alignment and structural comparison were used to identify the key amino acids involved in the interaction with activators and the structure difference between β1 and β2 subunits. Additionally, a series of potential β1-selective AMPK activators were identified by virtual screening using molecular docking. The retrieved hits were filtered on the basis of Lipinski’s rule of five and drug-likeness. Finally, 12 novel compounds with diverse scaffolds were obtained as potential starting points for the design of direct β1-selective AMPK activators.

## 1. Introduction

Kidney disease associated with diabetes is the leading cause of chronic kidney disease (CKD) and end-stage kidney disease worldwide and nearly one-third of patients with diabetes develop nephropathy [1]. As the incidence of both types 1 and 2 diabetes rises worldwide, diabetic nephropathy (DN) is likely become a significant health and economic burden for society [2]. Current therapy for diabetic nephropathy includes glycemic optimization using antidiabetics and blood pressure control with blockade of the renin-angiotensin system [3]. However, these strategies are slow but cannot reverse or at least stop the disease progression [4]. Although several clinical trials are currently in progress, there are still no drugs approved for the treatment of DN. Among these ongoing phase 3 clinical trials, atrasentan is still in progress, while bardoxolone methyl and paricalcitol failed to meet the primary endpoint or was terminated on safety concerns [4,5]. Recently, there has been renewed interest in the development of direct β1-selective Adenosine monophosphate-activated protein kinase (AMPK) activators that have the potential to treat diabetic nephropathy [6].

AMPK is master sensor of cellular energy and plays a critical role in the regulation of metabolic homeostasis [7]. AMPK is a heterotrimeric kinase comprised of a highly conserved catalytic α subunit and two regulatory subunits (β and γ) [8]. The α subunit possess a N-terminal serine/threonine catalytic kinase domain (KD) that is followed by an autoinhibitory domain (AID) and a C-terminal β subunit-binding domain [9]. The β subunit serves as a scaffold to bridge α and γ subunits that contains a glycogen binding domain (GBD) and a C-terminal domain [10]. The γ subunit is composed of a β subunit-binding region and two Bateman domains [11]. These seven subunits (α1, α2, β1, β2, γ1, γ2, and γ3) are encoded by separate genes, resulting in 12 different αβγ AMPK heterotrimers [12]. The distinct physiological functions of each AMPK isoforms are not fully understood, but derive from differential expression patterns among different tissues [13]. For instance, the α1 subunit appears to be relatively evenly expressed in kidney, rat heart, liver, brain, lung and skeletal muscle tissues, while the α2 subunit is mainly expressed in skeletal muscle, heart, and liver tissues [14]. Among the two known β subunits, β1 subunit is highly abundant in kidney as suggested by mRNA levels [6].

**Figure 1:**
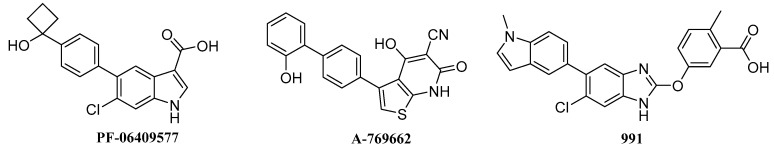
Structures of reported direct AMPK activators.

More recently, a direct AMPK activator PF-06409577 was reported to activate α1β1γ1 and α2β1γ1 AMPK isoforms with EC_50_ of 7.0 nM and 6.8 nM but was much less active against α1β2γ1/α2β2γ1/α2β2γ3 AMPK isoforms with EC50 greater 4000 nM [6]. Besides, compound PF-06409577 exhibited efficacy in a preclinical model of diabetic nephropathy. Compounds A-769662 and 991 possessed similar potency toward AMPK heterotrimers containing a β1 subunit as PF-96409577 [15]. On the other hand, an allosteric site of AMPK has been named allosteric drug and metabolite site (ADaM site) [16], which was constructed by the catalytic kinase domain (KD) of α subunit and the regulatory carbohydrate-binding module (CBM) of β subunit [13,17]. The three known direct AMPK activators (PF-06409577 [6], A-769662 [18], and 991 [19], Figure 1) all bound to the allosteric site and showed better potency for isoforms that contain the β1 subunit. This implies that the allosteric site can be used to design the selective activators of AMPK containing the β1 subunit.

The present study aims to investigate details of the domain structure and identify new potential β1-selective AMPK activators. Hence, sequence alignment and structural comparison were used to identify the key amino acids that are involved in the interaction with activators and structure difference between different subunits. Furthermore, molecular docking was performed for virtual screening to discover direct β1-selective AMPK activators. The screened retrieved hits were then subjected to several filters such as estimated activity and quantitative estimation of drug-likeness (QED) [20,21]. Finally, 12 compounds with diverse scaffolds were selected as potential hit compounds for the design of novel β1-selective AMPK activators. These findings provided a useful molecular basis for the design and development of novel β1-selective AMPK activators.

## 2. Results and Discussion

### 2.1. Sequence Alignment and Structural Comparison

**Figure 2:**
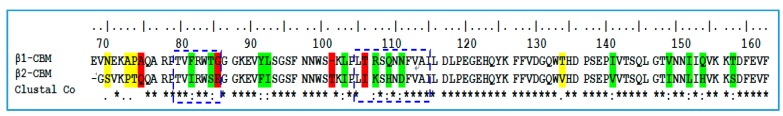
Sequence alignment of carbohydrate-binding module from the β1 and β2 subunits. Asterisks indicate positions that have a single, fully conserved residue. Colon (green) indicates conservation between groups of strongly similar properties. Period (yellow) indicates conservation between groups of weakly similar properties. Blank character (red) indicates conservation between groups of strongly different properties.

**Figure 3:**
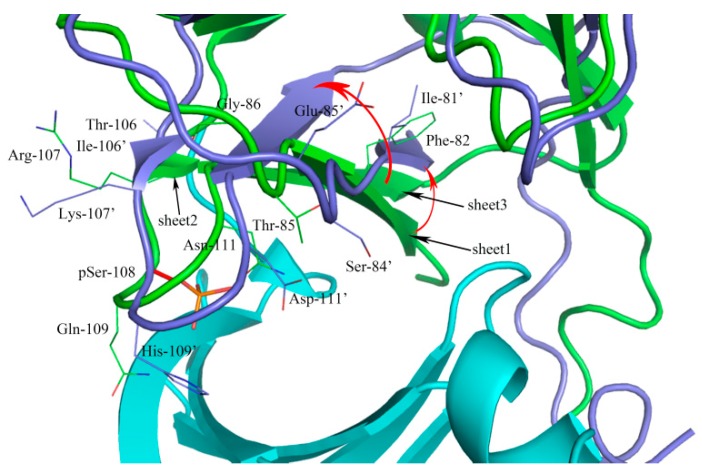
Structural comparison of the scope within 5 Å of the active site from β1 and β2 subunits. The α subunit was shown in cartoon and colored by the cyan. The β1 and β2 subunits were shown in cartoon and colored by green and blue, respectively. The sites with different amino acids were shown in line. The Ser-108 was shown in stick and colored by red.

To reveal the possible molecular mechanism for the selective potency of activators against the β1-isoform of AMPK, sequence and secondary structure elements comparison between carbohydrate-binding module of β1 and β2 subunits were investigated. As shown in Figure 2, the sequences that were boxed blue were located within the range of 5 Å of active site. Sequence alignment reveals that β1 and β2 subunits shares 77.1% sequence identity. As shown in Figure 3, superposition with the two subunits reveals a deflexion of sheet1 in β2 subunit as compared with β1 subunit. The Phe-82 of β1 subunit corresponded to Ile-81 in β2 subunit, as well as the Thr-85 to Ser-84, Gly-86 to Glu-85, which may account for the deflexion of sheet1 in β2 subunit. The large aromatic Phe residues and small Thr and Gly presented a binding surface more capable of accommodating ligand.

The sheet 2 torsion may attribute to the amino acid sequence differences of the sites of 106 and 107. The most notable is supposed to the Ser108 (red and stick), an autophosphorylation site, phosphorylated serine (pSer108) formed hydrogen bonds with Thr-21, Lys-29, Lys-31, His-109′, and Asn-111′ enhancing the ADaM site stabilization [22], and the phosphate group contributed to the binding of activators [23]. The Gln-109′ and Asn-111′ were mutated to His-109′ and Asp-111′, which abolished original hydrogen bonds and generated a large conformational change. We speculated that the above differences between β1 and β2 may affect the binding of activators to AMPk isoforms.

### 2.2. Parameter Setting for Molecular Docking

**Figure 4:**
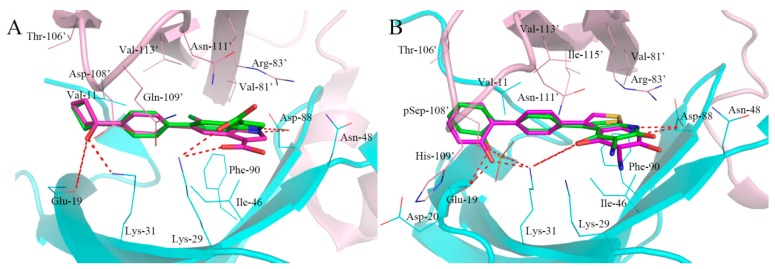
Conformation comparison of the original ligand from X-ray crystal (magenta and stick) and the conformation from Surflex-Dock result (green and stick). (**A**): PF-06409577; (**B**): A-769662. The indole moiety of PF-06409577 and terminal benzene ring of A-769662 generated a little deflection in compared with the original conformation. The hydrogen bond was labeled by red dashed lines.

Docking parameters, which exert an important influence on molecular docking-based virtual screening, were optimized in advance. The crystal structure of PF-06409577 bound to the α1β1γ1 AMPK isoform (PDB ID: 5KQ5) and A-769662 bound to the α2β1γ1 AMPK isoform (PDB ID: 4CFF) were chosen as the reference, the docking parameters were adjusted until the docked poses were as close as possible to the original crystallized structures. The ring flexibility was mainly considered in final optimized docking parameters according to the default settings. The overlay of the original ligand from X-ray crystal (stick and magenta) and the conformation from Surflex-Dock results (stick and green) were shown in Figure 4, in which the indole moiety of PF-06409577 and terminal benzene ring of A-769662 generated a little deflection and there was no effect on the interaction between compounds and the active site. The hydrogen bond interactions appeared consistent with the original ligands and the root mean square deviation (RMSD) between these two conformations are 0.53 and 0.56 Å, respectively. The molecular docking results indicated that the Surflex-Dock was reliable and could be used for the further virtual screening.

### 2.3. High-Throughput Virtual Screening Procedure

To identify new potent activators of AMPK, virtual screening was performed on the active sites as mentioned previously. A chemical library containing with 1,500,000 commercially available compounds (ChemDiv database) was docked to the molecular models of α1β1γ1 and α2β1γ1 AMPK isoforms in silico, respectively. Prior to docking, the ChemDiv database was split into eight subsets for molecular docking. About 600 top ranked compounds with high total-scores were screened and subsequently checked for their binding modes and interactions with the active site, especially the hydrogen bonds formed with the residuals of Asp-88, Lys-29, Lys-31, and Gly-19. Then the potential hit compounds were evaluated for their drug-likeness model scores using Lipinski’s rule of five (Table 1). Finally, six potential hits with new scaffolds could serve as activators for α1β1γ1 AMPK isoform and six for α2β1γ1 AMPK isoform were visually chosen from the top potential hits.

### 2.4. Analysis of Binding Mode of Activators for α1β1γ1 AMPK Isoform

**Figure 5:**
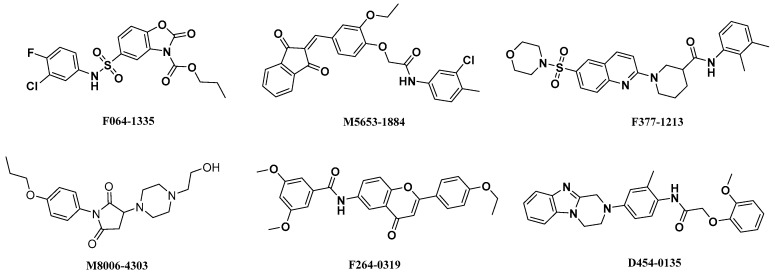
Structures of retrieved hits targeting α1β1γ1 AMPK isoform from ChemDiv database.

The structures of retrieved hits as activators of α1β1γ1 AMPK isoform are shown in Figure 5. Although these compounds possess different chemical scaffolds, they exhibit similar binding modes at the active site. Among these compounds, compounds F064-1335 and M5653-1884 possess higher docking scores, compounds M8006-4303 and F264-3019 have perfect drug-likeness model scores.

**Figure 6:**
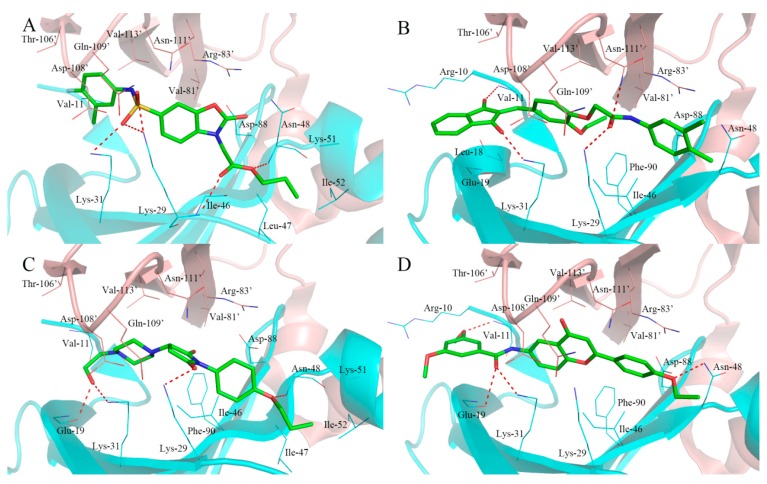
The binding modes of typical hit compounds for α1β1γ1 AMPK isoform. (**A**): F064-1335; (**B**): M5653-1884; (**C**): M8006-4303; (**D**): F264-0391. The α subunit was shown in cartoon and colored by cyan and the β subunit was shown in cartoon and colored by pink. The hydrogen bonds were labeled with red dashed lines.

As shown in Figure 6A, the compound F064-1335 with the highest docking score (10.50) formed several hydrogen bonds with active site residues. The two oxygen atoms of sulfonamide established a hydrogen bond network with the side chain of Lys-31, Lys-29, and Asn-111′. The carbonyl oxygen atom of the ester group formed two hydrogen bonds with the main chain of Lys-29, the anther oxygen atom of the ester group was bound to the main chain of Asn-48 by a hydrogen bond, which made the alkoxy group trend into a hydrophobic pocket formed by the Lys-51, Ile-52, Val-62, and Leu-47. In addition, the carbonyl oxygen of benzoxazolone ring formed a hydrogen bond with the side chain of Arg-83′.

The compound M5653-1884 with a considerable docking score (10.24) and the bind mode is shown in Figure 6B. Four hydrogen bonds were observed between the compound and the active site residues. One carbonyl oxygen atom of 1,3-indandione formed hydrogen bond with the side chain of Lys-31, another carbonyl oxygen atom formed a hydrogen bond with the main chain of Val-11. The carbonyl oxygen atom of the amide group showed hydrogen bond interactions with the side chain of Lys-29 and Asn-111. In addition, there was a hydrophobic effect with the side chain of Ile-46, Asn-48, Asp-88, and Phe-88.

As shown in Figure 6C, the compound of M8006-4303 exhibited similar binding mode as PF-06409577. The ethanol group attached to the piperazine group participated in two hydrogen bond interactions with the side chain of Gly-19 and Lys-31. The carbonyl oxygen atoms of pyrrolidine-2,5-dione formed a hydrogen bond interaction with the side chain of Lys-29. In addition, the oxygen atom of oxygen butyl associated with the benzene ring accepted a hydrogen bond from the main chain of Asn-48. Within the cavity of the active site, Ile-47, Asn-48, Lys-51, and Ile-52 probably generated a hydrophobic effect.

The binding mode of compound F264-3091 with a prefect drug-likeness score (1.00) was shown in Figure 6D. The oxgen atom of an oxyethyl group on the benzene ring participated in a hydrogen bond with the main chain of Val-11. The carbonyl oxygen atom of the amide group showed two hydrogen bonds with the main chain of Gln-19 and side chain of Lys-31. In addition, one hydrogen bond was formed between the side chain of Asn-48 and the oxyethyl group connected with flavone B-ring while the B-ring showed a stacked cation-π interaction with the side chain of Val-83′.

### 2.5. Analysis of Binding Mode of Activators for α2β1γ1 AMPK Isoform

**Figure 7:**
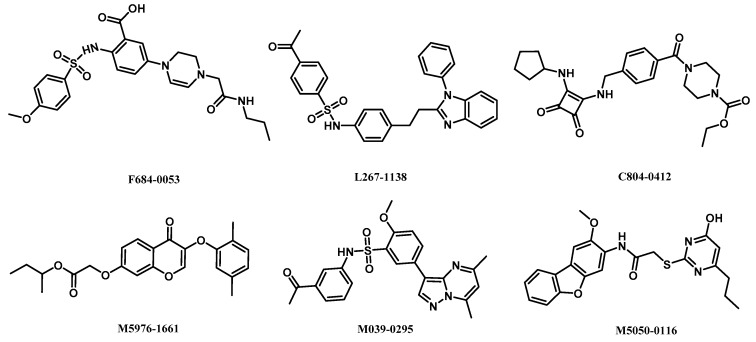
Structures of retrieved hits targeting α2β1γ1 AMPK isoform from ChemDiv database.

**Figure 8:**
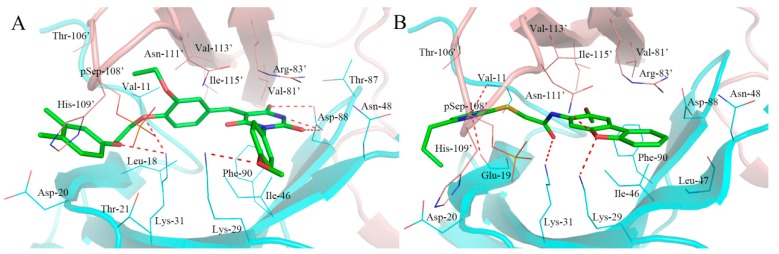
The binding modes of typical activators for α2β1γ1 AMPK isoform. (**A**): M2958-7438; (**B**): M5050-0116. The α subunit was shown in the illustration and colored cyan and the β subunit was shown in the illustration and colored pink. The hydrogen bonds were labeled with red dashed lines.

The chemical structures of six compounds as activators of α2β1γ1 AMPK isoform are shown in Figure 7. The molecular docking results indicated that all the compounds possess higher docking scores than A-769662 and 991. The binding modes of the representative compound M2958-7438 and M5050-0116 in the active site of α2β1γ1 AMPK isoform are shown in Figure 8.

As shown in Figure 8A, six hydrogen bonds were formed between the compound M2958-7438 and active site residues, in which the barbituric acid ring formed three hydrogen bonds with the side chain of Asp-88, making prominent contributions to the high docking score (10.04). The oxygen atom of the anisole associated with the barbituric acid ring accepted a hydrogen bond from the side chain of Lys-29, and two oxygen atoms in the linker participated in two hydrogen bonds with the side chain of Lys-31. In addition, the barbituric acid ring generated a stacked cation-π interaction with the side chain of Arg-83′.

The compound M5050-0116 with a docking score of 9.27 and formed four hydrogen bonds with Val-11, Leu-18, Lys-29, and Asn-111′. As shown in Figure 8B, the Lys-29 of α subunit and Asn-111′ of β subunit, simultaneously coordinated the oxygen atom of dibenzofuran with hydrogen bonds. The hydroxyl group attached on pyrimidine anchored in a suitable geometry and formed two hydrogen bonds with the main chain of Glu-19 and the side chain of Val-11. Additionally, the compound M5050-0116 exhibited hydrophobic interactions with several residues, which formed a hydrophobic pocket including Ile-46, Leu-47, Asn-48, Asp-88, and Phe-90.

### 2.6. Biological Activities

**Figure 9:**
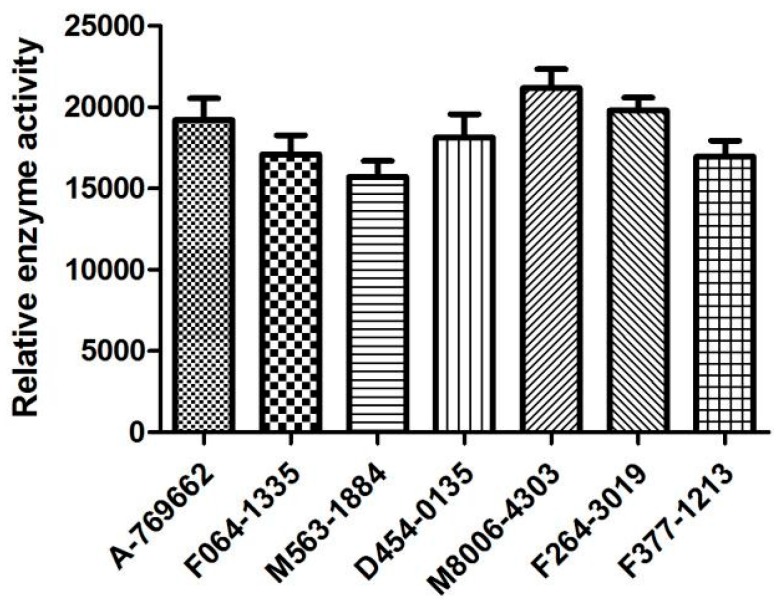
Activation of AMPK (α1β1γ1 isoform) by the screened compounds was measured using Elisa Kit.

The six screened compounds based on α1β1γ1 AMPK isoform were evaluated for activities against AMPK (α1β1γ1 isoform) at a dosages of 2 µM. A-769662, a known β1-selective AMPK activator, was used as a control. The preliminary in vitro assay (Figure 9) indicated that most of the selected α1β1γ1 AMPK activators displayed promising activation potency against α1β1γ1 AMPK isoform. Compounds D454-0135 and F264-3019 displayed comparable activation activity against AMPK in the comparison with the known β1-selective AMPK activator A-769662. Morever, compounds M8006-4303 and F264-3019 showed stronger activation activities against α1β1γ1 AMPK than A-769662. Compound M563-1884 with a higher logP value and showed a relatively low activation activity among the assayed compounds. This implies that the lipophilicity may play an important role in the bioavailability for the compound.

## 3. Materials and Methods

### 3.1. Sequence Alignment and Structural Comparison

Sequence alignment is an essential method for similarity/dissimilarity analysis of protein, DNA, or RNA sequences [24]. The software used for sequence alignment tasks include HAlign, BioEdit, EMBL-EBI, T-Coffee, and CLUSTAL [25,26]. The crystal structure data of AMPK (α1β1γ1: 5KQ5, 4QFG; α1β2γ1: 4REW and α2β1γ1: 4CFF) were obtained from RCSB Protein Data Bank [6,8,13,19], as well as the amino acid sequences of carbohydrate-binding module. The amino acid sequences of carbohydrate-binding module (CBM) on β subunits were used to study the differences. The sequence alignment between α1β1γ1 isoform (PDB: 4QFG) and α1β2γ1 (PDB:4REW) isoform was performed and edited using BioEdit software (version 7.1.8) [27], which is a user-friendly biological sequence alignment editor and analysis program. The crystallographic structures of AMPK for molecular docking studying were added to the hydrogen atoms and the charge was given to the Gasteiger-Huckel. The crystal structures comparison was conducted by Sybyl X 2.1 (Tripos Associates Inc., S.H. R.: St. Louis, MO, USA.) [28] and the binding modes were generated by PyMOL V0.99 (Schrödinger, New York, NY, USA.) [29]. The polar hydrogen atoms were added to the crystal structures of the AMPK via the biopolymer module and the Gasteiger-Huckel charges were loaded on the atoms of proteins. The protein peptide backbones were shown in cartoon and colored by different colors, the side chains of the nonconservative amino acids were shown in line and colored by chain.

### 3.2. Molecular Docking

The virtual screening and molecular docking studies were performed using Surflex docking module in Sybyl X 2.1. There were still some deficiencies due to the fact that the receptor was regarded as a rigid structure. Therefore, it was essential to optimize the docking parameters, the co-crystallized ligand was extracted and re-docked into the active site of the AMPK with the varied parameters, and then the conformation of the original ligand and the re-docking ligand were compared. The binding site was defined as a sphere containing the residues that stay within 5 Å from the co-ligand. The maximum conformations per fragment and maximum number of rotatable bonds per molecule were 20 and 100, respectively. Furthermore, the options for pre-dock minimization and post-dock minimization of molecules were omitted, while other parameters were set as default options. The top 20 conformational poses were selected according to the docking score. Dock scores were evaluated by Consensus Score (CScore), which integrates the strengths of individual scoring functions combine to rank the affinity of ligands bound to the active site of a receptor.

### 3.3. High-Throughput Virtual Screening

High-throughput virtual screening was regarded as an important tool to identify novel lead compounds suitable for specific protein targets [30], and the screened compounds can be easily obtained from commercial sources for biological evaluation as well [31]. The ChemDiv database was supplied by Topscience Co. (Shanghai, China), which includes 1,500,000 compounds was employed for virtual screening through Surflex docking module in Sybyl X 2.1. To accelerate virtual screening, the maximum quantity of conformations was reduced from 20 to 10, the maximum quantity of rotatable bonds was decreased from 100 to 50, and the top six conformations were collected. The same as the molecular docking studies, the default optimization of molecules before and after the docking was canceled. Other parameters were kept as default values. Compounds PF-06409577 and A-769662 were severed as reference molecules, respectively. The compounds with the docking score (≥8.0) were extracted for further analyzing the interactions between ligand and active site, to this end, 100 compounds were collected to calculate the drug-likeness model score. Drug-likeness model scores were computed for hit compounds using the MolSoft software (MolSoft, San Diego, CA, USA) [32].

### 3.4. The In Vitro Activation Assay

The in vitro preliminary kinase assays human α1β1γ1 AMPK were carried out according to the previous experimental method [33]. The screened compounds and α1β1γ1 AMPK isoform were provided by Topscience Co. (Shanghai, China) and Huawei Pharmaceutical Co. Ltd. (Shanghai, China), respectively. Generally, each of the evaluated compounds was dissolved in 10% Dimethyl sulphoxide at 10 μM and diluted to a required concentration with buffer solution. Then, 5 μL of the dilution was added to a 30 μL kinase assay buffer and 5 μL AMPK isoform per well. The solution was mixed at 0 °C for 30 min. Next, 5 μL of AMARA petide and 5 μL Adenosine triphosphate (ATP) were added to the well. The enzymatic reactions were conducted at 30 °C for 30 min. The AMPK activity was determined by quantifying the amount of ATP remaining in assay solution with Kinase-Glo Plus luminescent kinase assay kit (Promega, Madison, WI, USA). The luminescent signal is correlated with the amount of ATP present, while inversely correlated with the kinase activity. The mean values from three independent experiments were used for the expression of relative activities. A-769662, a β1-selective AMPK activator reported by Abbott laboratories, was used as a control.

## 4. Conclusions

In summary, the sequence alignment and structural comparison was performed to identify the AMPK domain structure detail, which provides a molecular basis of selective AMPK activators on β1-containing isoforms. The key amino acid residues (Phe/Ile82, Thr/Ser85, Gly/Glu86, Thr/Ile106, Arg/Lys107, Gln/His109, Asn/Asp111) may contribute to the selectivity and provide a foundation for structure-based design of new direct β1-selective AMPK activators. Furthermore, the structure-based virtual screening workflow for the identification of selective activators of AMPK (α1β1γ1 and α2β1γ1) was established and six potential hit compounds for α1β1γ1 isoform and α2β1γ1 isoform were obtained, respectively. The preliminary assay indicated that most of the selected α1β1γ1 AMPK activators displayed promising activation potency. Overall, these findings revealed extensive interactions of activators and AMPK for rational design of novel selective AMPK activators. Further in vitro testing of retrieved hits is still in progress in our laboratory.

## Figures and Tables

**Table 1:** The docking scores and drug-likeness model scores of selected activators for AMPK (α1β1γ1 and α2β1γ1).

Isoforms	Compound No.	Total-Score	Crash	Polar	Similarity	Number of HBA/HBD	MolLog P	Drug-Likeness Model Score
AMPK (α1β1γ1) activators	F064-1335	10.50	−2.35	3.54	0.44	6/1	3.79	−0.16
	M5653-1884	10.24	−1.43	2.90	0.50	5/1	6.07	0.22
	D454-0135	10.20	−1.58	3.41	0.44	6/2	4.03	−0.31
	M8006-4303	10.07	−1.83	4.29	0.58	6/1	1.01	1.02
	F264-3019	9.93	−1.39	3.19	0.46	6/1	5.44	1.00
	F377-1213	10.03	−1.43	1.32	0.44	6/1	4.12	0.16
	PF-06409577	7.29	−0.07	3.08	0.93	3/3	3.80	0.71
AMPK (α2β1γ1) activators	L267-1138	10.96	−2.46	2.78	0.52	4/1	6.05	−0.08
	F684-0053	10.60	−2.77	4.31	0.54	7/3	2.04	0.54
	C804-0412	10.15	−3.27	3.39	0.54	5/2	2.53	1.00
	M5976-1661	9.46	−0.92	1.32	0.46	6/0	4.84	0.62
	M039-0295	9.35	−1.61	1.48	0.50	6/1	2.66	−0.20
	M5050-0116	9.27	−1.35	2.82	0.49	7/2	5.13	0.38
	A-769662	7.44	−1.46	1.26	0.93	5/3	3.46	0.30
	991	8.38	−0.96	4.08	0.73	4/2	5.38	0.41
